# Tolerance and Reduction of Chromium(VI) by *Bacillus* sp. MNU16 Isolated from Contaminated Coal Mining Soil

**DOI:** 10.3389/fpls.2017.00778

**Published:** 2017-05-22

**Authors:** Neha Upadhyay, Kanchan Vishwakarma, Jaspreet Singh, Mitali Mishra, Vivek Kumar, Radha Rani, Rohit K. Mishra, Devendra K. Chauhan, Durgesh K. Tripathi, Shivesh Sharma

**Affiliations:** ^1^Department of Biotechnology, Motilal Nehru National Institute of Technology AllahabadAllahabad, India; ^2^Centre for Medical Diagnostic and Research, Motilal Nehru National Institute of Technology AllahabadAllahabad, India; ^3^Department of Biotechnology, Himalayan Institute of Biosciences, Swami Rama Himalayan UniversityDehradun, India; ^4^D D Plant Interdisciplinary Research Laboratory, Department of Botany, University of AllahabadAllahabad, India

**Keywords:** *Bacillus subtilis*, chromium(VI), propidium iodide, flow cytometry, fluorescence microscopy

## Abstract

The bacterium MNU16 was isolated from contaminated soils of coal mine and subsequently screened for different plant growth promoting (PGP) activities. The isolate was further identified by 16S rRNA sequencing as *Bacillus subtilis* MNU16 with IAA concentration (56.95 ± 0.43 6μg/ml), siderophore unit (9.73 ± 2.05%), phosphate solubilization (285.13 ± 1.05 μg/ml) and ACC deaminase activity (116.79 ± 0.019 μmoles α-ketobutyrate/mg/24 h). Further, to evaluate the metal resistance profile of bacterium, the isolate was screened for multi-metal resistance (*viz.* 900 mg/L for Cr, 600 mg/L for As, 700 mg/L for Ni and 300 mg/L for Hg). Additionally, the resistance pattern of *B. subtilis* MNU16 against Cr(VI) (from 50 to 300 mg/L) treatments were evaluated. An enriched population was observed at 0–200 mg/L Cr(VI) concentration while slight reductions were observed at 250 and 300 mg/L Cr(VI). Further, the chromium reduction ability at 50 mg/L of Cr(VI) highlighted that the bacterium *B. subtilis* MNU16 reduced 75% of Cr(VI) to 13.23 mg/L within 72 h. The localization of electron dense precipitates was observed in the TEM images of *B. subtilis* MNU16 which is might be due to the reduction of Cr(VI) to Cr(III). The data of fluorescence microscopy and flow cytometry with respect to Cr(VI) treatments (50–300 mg/L) showed a similar pattern and clearly revealed the less toxic effect of hexavalent chromium upto 200 mg/L Cr(VI) concentration. However, toxicity effects were more pronounced at 300 mg/L Cr(VI). Therefore, the present study suggests that the plant growth promoting potential and resistance efficacy of *B. subtilis* MNU16 will go a long way in developing an effective bioremediation approach for Cr(VI) contaminated soils.

## Introduction

Soil contamination and degradation are important issues from both environmental and agricultural points of view. The continual anthropogenic activities, extensive use of chemical fertilizers, pesticides and rapid industrialization are responsible for increasing environmental pollution (Aktar et al., 2009; Singh et al., 2015, 2017; Tripathi et al., 2016a). These activities are major causes of soil degradation and contamination and thus pose a great challenge for food safety and public health (Qishlaqi and Moore, 2007; Tripathi et al., 2012, 2015, 2016b). Soils of mining sites have extremely high percentage of heavy metals such as chromium, lead, cadmium, and arsenic which cause destruction of soil properties such as pH, nutrient content and quality (Ladwani et al., 2012; Hansda et al., 2014); thereby, significantly affecting the fertility of surrounding soil, associated microbial community, vegetation status as well as pose several health risks at different levels of food chain through a well-established process of bioaccumulation (Wuana and Okieimen, 2011; Upadhyay et al., 2016).

Heavy metals are the most dominant form of contamination found in the waste dump sites of mines and their remediation is considered to be a technically difficult task due to their recalcitrant nature (Wuana and Okieimen, 2011). There are a number of traditional and conventional technologies which are widely acceptable to remediate metal contaminated soils. However, these approaches are time consuming and usually generate complex secondary waste. In this context, our major concern is to reclaim and restore soil properties through implementation of situation-specific technique(s) that permit the best possible decontamination at the lowest potential cost with modest or no side effects. The application of plant–microbe interaction for the remediation of contaminated soil is an important and well-adapted technology (Hansda et al., 2014). Plant growth is regulated by soil fertility which influences the physical (e.g., soil structure), chemical (e.g., soil pH, organic matter, C: N ratio) and biological (e.g., microbial diversity in soil) properties of soil. On the other hand, most of the time, adverse conditions of degraded soils results in the destruction of aboveground vegetation. Rhizobacteria generally colonize root and enhance plant growth in degraded soils, as they can alleviate biotic and abiotic stress by releasing phytohormones (e.g., IAA, ethylene), solubilizing minerals and producing iron chelating compounds (e.g., siderophores) (Ahemad, 2015). Several studies have reported the utilization of plant growth promoting (PGP) rhizobacteria for the bioremediation of heavy metals including *Bacillus* sp., *Pseudomonas* sp. etc. (Samuel et al., 2013; Sobariu et al., 2016; Ndeddy Aka and Babalola, 2016).

Among various heavy metals, chromium (Cr) is one of the most common pollutants originating mainly from industrial effluents, mining practices, petroleum refining industries and use of pesticides (Oliveira, 2012). In nature, chromium mainly exists in trivalent [Cr (III) form and found naturally in environment. Hexavalent (Cr (VI)] is mainly released in environment through various anthropogenic activities and imparts toxic effects on microorganisms, human beings and soil fertility (Oliveira, 2012; Tripathi et al., 2012; Ahemad, 2015). It is well documented that bacterial composition is greatly affected by soil type, its characteristics and stress factors which restrict the bacterial growth in soil (Upadhyay et al., 2016). Only a few bacteria may adapt to these stressed environments and proliferate. The transition into this physiological state and recovery were found to be correlated with the environmental stress equivalent to pH, temperature and bioavailability of minerals and nutrients (Olszewska et al., 2016).

The behavior of micro-organisms toward Cr(VI) was studied previously which demonstrated the significant alleviative impact of many microorganisms, including strains of *Pseudomonas, Enterobacter, Bacillus*, and *Shewanella* (Samuel et al., 2013; Ahemad, 2015). Microorganisms were found to reduce hexavalent chromium by different mechanisms either by utilizing the hexavalent chromium as final electron acceptor or by secreting certain soluble enzymes (Samuel et al., 2013; Ahemad, 2015). However, the availability of efficient hexavalent chromium reducing microorganisms is a crucial requirement for the bioremediation of soils contaminated with hexavalent chromium. With this background, the present study is envisaged to isolate and select the most resistant bacterial isolate from the contaminated soils with potential to reduce hexavalent chromium. In addition, the bacterial growth and morphological modifications were examined against various Cr(VI) treatments using modern techniques so that it can be utilized further for the effective bioremediation in associated with the enhancement in plant growth promotion when grown in contaminated soil.

## Materials and Methods

### Isolation and Characterization of Bacteria for PGP Traits

Samples were collected in triplicates from the degraded soils of coal mining site of Uttar Pradesh. Nutrient agar medium was used to isolate bacterial colonies by serial dilution technique and plates were observed after 24 h of incubation. Colonies were further purified by streak plate and preserved at 4°C for further experiments.

Plant growth promoting characteristics of isolates were further identified by the standard procedure discussed below. The bacterial isolates were screened for indole-3-acetic acid (IAA) production using L-tryptophan and quantified colorimetrically by Salkowski method (Loper and Scroth, 1986). The production of siderophore was initially screened on Chrome Azurol S (CAS) agar medium (Schwyn and Neilands, 1987) and further estimated quantitatively by CAS shuttle assay as per described by Payne (1994). An equal volume of culture supernatant was mixed with CAS reagent and absorbance was measured at 630 nm against the reference containing equal volume of uninoculated medium and CAS reagent. Percentage siderophore produced was calculated by using the formula:

%siderophoreunits = (Ar-As/Ar) × 100

Where Ar = absorbance of the reference and As = absorbance of the sample.

Bacterial isolates were further screened for phosphate solubilizing potential first qualitatively by growing isolates on Pikovskaya’s medium, and isolates showing clear zone were selected as potential phosphate solubilizer (Premono et al., 1996). Bacterial isolates that have the highest solublization index were selected for the quantitative analysis done by using Pikovaskaya Broth and estimated spectrophotometrically using chlorostannous-reduced-molybdo-phosphoric acid method (Yadav and Verma, 2012).

The ACC deaminase activity of the isolates was further determined by the method of Saleh and Glick (2001). The concentration of α- ketobutyrate produced by the action of ACC deaminase was determined spectrophotometrically and calculated with the help of standard curve. The amount of α- ketobutyrate produced is expressed as μ moles α-ketobutyrate/mg/24 h.

### Molecular Characterization of Potential Isolate

The genomic DNA was isolated from the bacteria by enzymatic method using phenol/chloroform (Maniatis et al., 1982). The DNA isolated was resuspended in TE buffer (pH 8.0) and amplified by PCR to obtain the 16S rRNA sequences. Universal primers, 8F (5′-AGAGTTTGATCCTGGCTCAG-3′) and 1492R (5′-AAGGAGGTGATCCAGCCGCA-3′) were used for DNA amplification. The PCR product was purified using QIAquick PCR Purification Kit (Qiagen). Purified PCR fragments were directly sequenced with Applied Biosystems, 3500XL Genetics Analyzer using the manufacturer’s instructions. Chromas Lite 2.0 software was used to read the sequences and the sequences were then aligned with the previous sequences available in the GenBank database by BLAST tool. A phylogenetic tree was created by the use of multiple sequence alignment tool, ClustalW and MEGA 6.06 software.

### Screening of Bacterial Isolates for Chromium(VI) Resistance

The selected bacterium was further screened for its resistance to chromium (Cr VI) by agar well diffusion method (Hassen et al., 1998). In addition the bacterium was also screened for resistance to various metals including Iron (Fe), Copper (Cu), Arsenic (As), Mercury (Hg), Cadmium (Cd) and Nickel (Ni). Various concentrations of metals were prepared by using their salts as: K_2_Cr_2_O_7_ for Cr(VI), NaAsO_2_ for As, HgCl_2_ for Hg, CdCl_2_ for Cd, FeCl_3_ for Fe and Ni(NO_3_)_2_ for Ni in distilled water and sterilized by autoclaving. Sterile MHA plates were prepared and spreaded with the overnight grown bacterial culture. After this, approximate 7 mm diameter wells were punched with a sterile borer and filled with metals (100 μl) with various concentrations to determine the value of MIC. The plates were then incubated at 30°C for 24–48 h and zones around the wells were measured. Metal concentration which gave a clear zone of 1 mm or less than 1 mm was considered as the MIC and the bacterium resistant for that particular concentration (Rani et al., 2010).

### Screening of Isolate for Cr (VI) Reduction

The ability of bacterial isolate to reduce Cr(VI) into less toxic form was analyzed using diphenylcarbazide (DPC) method by estimating the decrease in concentration of hexavalent chromium (Zahoor and Rehman, 2009). The 24 h old grown culture was inoculated in 100 ml of LB broth containing 50 mg/L of Cr(VI) as K_2_Cr_2_O_7_ at 30°C to estimate the chromium reduction. The samples were collected by centrifugation at 10,000 rpm for 10 min. The remaining concentration of Cr(VI) in the supernatant was determined by measuring the absorbance of Cr(VI)-DPC complex at 540 nm using spectrophotometer at various time intervals. The percentage reduction of Cr(VI) was calculated by using the following formula:

$$\mathrm{Cr}(\mathrm{VI})\mathrm{reduction}(\%)=\frac{A-B}{B}\times 100$$

Where A- Absorbance of control; B- Absorbance of sample.

### Transmission Electron Microscopy (TEM) Analysis

The bacterial strain was analyzed through the TEM to confirm the presence and accumulation of hexavalent chromium inside the cell and to detect the effect of metal on the bacterium. Samples were prepared according to the method described by Bano et al. (2013) with slight modifications. The bacterial cell without any treatment served as control and various concentrations of Cr(VI) were taken as treatments for TEM analysis. The 48 h grown bacterial culture was harvested and pellet was washed with 1 M phosphate buffer saline (PBS) buffer. The cells were fixed using fixative (2.5% glutaraldehyde and 2% paraformaldehyde in 1 M PBS) for 6 h at 4°C and then washed three times with PBS to remove the fixative solution. The pellet was then suspended in 1 M PBS buffer and sent to SAIF, AIIMS, New Delhi for TEM analysis.

### Assessment of Physiological Changes in Bacteria under Different Cr(VI) Treatments

#### Culture Preparations

The bacteria were inoculated in nutrient broth medium at 28–30°C overnight under continuous shaking. Samples were collected from the bacterial suspension at exponential phase and the concentration was adjusted to nearly 2 × 10^6^ bacteria/ml (Walberg et al., 1997). The pellet of bacterial cells were then collected by centrifugation at 8000 rpm for 5 min, washed thrice with 0.1 M phosphate buffered saline (PBS) and resuspend the pellet in PBS to reach an optical density of 0.1 at 600 nm.

#### Exposure to Chromium Treatment

The bacterial cells were analyzed for their resistance pattern against various concentrations of chromium (VI) by studying CFU pattern and performing growth profile study by UV spectrometry, FM and FCM. The metal stress was generated by method described by Boswell et al. (1998). Potassium dichromate (K_2_Cr_2_O_7_) salt solution was prepared for generating chromium stress at concentration starting from 50, 100, 150, 200, 250, and 300 mg/L and the bacterial suspension was then exposed to different concentration of Cr(VI). A control was prepared without any metal exposure. The resistance pattern and bacterial responses in the presence of various Cr(VI) treatments were studied after 12 h of incubation.

#### Fluorescence Spectroscopy and Flow Cytometric Measurements

The bacterial suspension was further stained with PI, a nucleic acid staining dye, and analyzed through FM and FCM. After treatment, 10 μl PI (40 μg/ml) was added to 200 μl of bacterial suspension in the centrifuge tube and incubated for 15 min in dark to allow staining of the cells. The cells were immediately analyzed after the incubation period by FM and FCM.

Flow cytometry study was carried out using a BD Accuri^TM^ C6 Flow Cytometer with a red laser of 14.7 mW output and a constant wavelength excitation of 640 nm and a blue laser of 20 mW with excitation at a wavelength of 488 nm. The FCM instrument detects forward scatter (FSC) and side scatter (SSC) and is equipped with four different types of fluorescence detectors with optical filters. The red color (FL2, 585 nm; PE/PI) fluorescence detector was used for the current flow cytometric study. The optical detectors employed to collect the scattered laser light and fluorescent emissions, and electronics digitize these signals for computational analysis. The light scatter data gives a basic idea regarding cells relative size, and morphology. The fluorescence data reveals the cells’ auto fluorescence and/or labeling with fluorescent dyes, which can help characterize bacteria, resolve them from electronic noise and debris, and indicate cell viability and vitality. A detection limit of 10,000 bacteria was set for each sample and all experiments were conducted in triplicate.

### Statistical Analysis

The data obtained from the study were statistically analyzed and presented with the appropriate standard deviation from the data obtained in triplicates. The data were standardized by one-way analysis of variance (ANOVA). The least significant differences among means were compared at *P* ≤ 0.05 significance level. The data analysis and preparation of graphs were done by using the software OriginPro 8. 3.

## Results

### Isolation and Characterization of Bacteria to Test Plant Growth Promoting Activity

A total of 26 bacterial isolates were isolated from the degraded soils of coal mines and further characterized for their growth promoting characteristics. Bacterial isolate MNU16 was found to be positive for all screened PGP characteristics and proved to be the most effective producer of maximum growth promoting substances and therefore, selected for further studies. Five isolates were recorded as being efficient IAA producers, out of which highest IAA production was shown by isolate MNU16 with an IAA concentration of 56.95 ± 0.436 μg/ml (**Table 1**) after 48 days of incubation.

**Table 1:** Details of plant growth promoting traits produced by *Bacillus subtilis* MNU16 strain.

Plant growth promoting characteristics	Results
IAA production (μg/ml)	56.95 ± 0.436ˆa
Siderophore production (%)	9.73 ± 2.05ˆb
Phosphate solubilization (μg/ml)	285.13 ± 1.05ˆc
ACC deaminase activity (μ moles α-ketobutyrate/mg/24 h)	116.79 ± 0.019ˆe
Cr(VI) reduction %	75.39% ± 0.97ˆdc

*IAA, Indole 3-acetic acid. Results are average of data in triplicates ± SD (standard deviation). Mean with different letters in the same column differ significantly at *P* ≤ 0.05 (One-way ANOVA) significance level.*

Siderophore production was further checked by plate assay and the isolates that were able to produce an orange halo zone around the bacterial colony were selected as siderophore producers. The production of siderophore by isolate MNU16 was first observed in plate assay and then further quantified for siderophore production. On quantifying, it was observed that the isolate MNU16 produced 9.73 ± 2.05% siderophore in succinate medium (**Table 1**).

**FIGURE 1:**
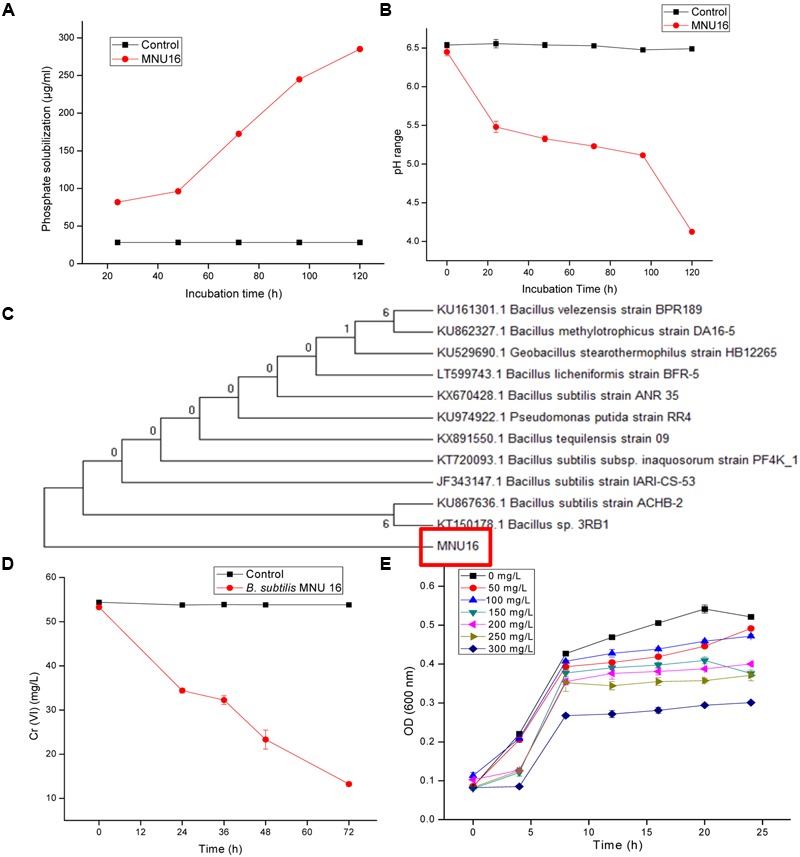
**(A)** Quantitative solubilization of phosphate by *Bacillus* sp. MNU16. **(B)** Lowering of pH in the broth due to P-solubilizing activity of *Bacillus* sp. MNU16. Data are average ± standard deviation of experiments in triplicate. **(C)** Phylogenetic tree, based on 16S ribosomal RNA gene sequences, showing relationships between MNU16 and other *Bacillus* sp. **(D)** Chromium [Cr (VI)] reduction by *Bacillus subtilis* MNU16 at 120 rpm and 30°C. **(E)** Growth of *B. subtilis* MNU16 in the presence of various concentration of chromium at various time intervals. Data are average ± standard deviation of experiments in triplicate.

The bacterial strain MNU16 was also assessed for ACC deaminase activity, and 116.79 ± 0.019 μ moles α-ketobutyrate/mg/24 h (**Table 1**) activity was recorded. The phosphate solubilization activity of the bacterial strain MNU16 was observed by the presence of clear halo zone around the Pikovskaya’s agar plate. It was also observed that the phosphate solubilization zone increased with increasing incubation time around the bacterial colony. The bacterial isolate was used to further quantified for phosphate solubilization potential. A significant increase in phosphate solubilization was observed concomitant with the decrease in pH, as the period of incubation increased from 24 to 120 h (**Figures 1A,B**). The quantitative estimation of phosphate solubilization is presented in **Figure 1A**. The bacterium was found to solubilize 81.75 μg/ml of phosphate after 24 h of incubation and maximum solubilization (285.13 ± 1.05 μg/ml) was recorded after 120 h of incubation. A continuous decline in the medium pH was observed from 6.5 to 4.1 which is presented in **Figure 1B**. It was clearly observed that the initial pH of the medium was 6.5 which decreased to 5.5 in 24 h and after 120 h, the pH of the medium was found to be 4.1, which is due to the production of several organic acids that are responsible for phosphate solubilization.

### Identification of Isolate through Molecular Characterization

On the basis of 16S rRNA gene sequence, isolate MNU16 showed maximum similarity with *Bacillus* spp. The *B. subtilis* MNU16 sequences were submitted to Genbank database with Accession No. KY012360. A phylogenetic tree (**Figure 1C**) was constructed using MEGA 5.0 by comparing it with other 16S rRNA sequences of related species and the isolate showed maximum similarity with *Bacillus* sp. (Accession No. KT150178.1).

### Identification of Minimal Inhibitory Concentration of Cr(VI)

The bacterial isolate was screened for chromium (Cr) and resistance to other heavy metals (As, Fe, Hg, Ni, Cd, and Cu) by agar well diffusion method (Supplementary Figure S1). The bacterial isolate MNU16 was found to be resistant at 900 mg/L chromium concentration. The MIC for Cd was recorded upto 150 mg/L, 300 mg/L for Hg, 600 mg/L for As, 700 mg/L for Ni, 1000 mg/L for Fe and 500 mg/L for Cu, respectively (Supplementary Figure S1). The order of toxicity of metals for the strain MNU16 was observed as highest for cadmium>mercury>arsenic>nickel>chromium>iron.

### *Bacillus subtilis* MNU16 under Different Chromium Treatments

The growth profile of bacterium *B. subtilis* MNU16 was performed at different Cr(VI) treatments to study the growth pattern of isolate at different time intervals. In addition, the isolate *B. subtilis* MNU16 was also studied for its potential to reduce hexavalent chromium [Cr(VI)] in nutrient broth medium having initial chromium concentration of 50 mg/L (**Figure 1D**). Further, it was observed that the isolate reduced the concentration of chromium upto 13.23 mg/L within 72 h of incubation at 30°C and shaking at 120 rpm (**Figure 1D**). The growth kinetics of bacterial isolate in the presence of chromium is shown in **Figure 1E** which illustrated that the bacterium shows higher growth at chromium concentrations 50, 100, 150, and 200 mg/L. However, the absorbance reduced at higher concentration and bacterial growth got affected at 250 and 300 mg/L as shown in **Figure 1E**.

### Physiological Modulations in *Bacillus subtilis* MNU16 under Different Cr Treatments

**FIGURE 2:**
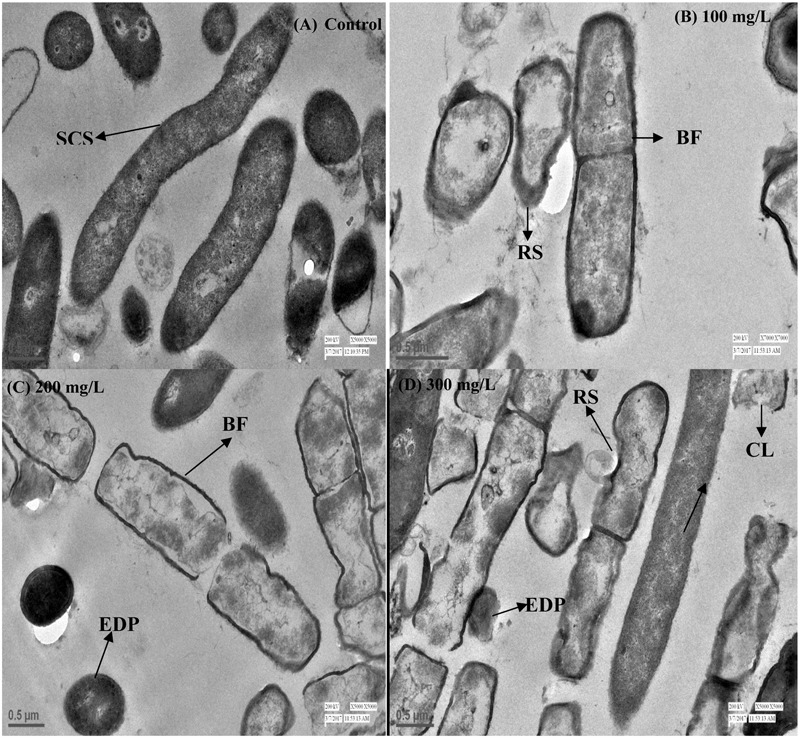
**Transmission electron microscopy (TEM) images of cross sectioned *B. subtilis* MNU16 cells at (A)** control (without treatment). **(B)** *B. subtilis* MNU16 cells at 100 mg/L of Cr(VI) **(C)** *B. subtilis* MNU16 cells at 200 mg/L of Cr(VI) and **(D)** at 300 mg/L of Cr(VI) concentration. Arrows signifies SCS, smooth cell surface; RF, rough surface; BF, binary fission; EDP, electron dense precipitates; CL, cell lysis.

**FIGURE 3:**
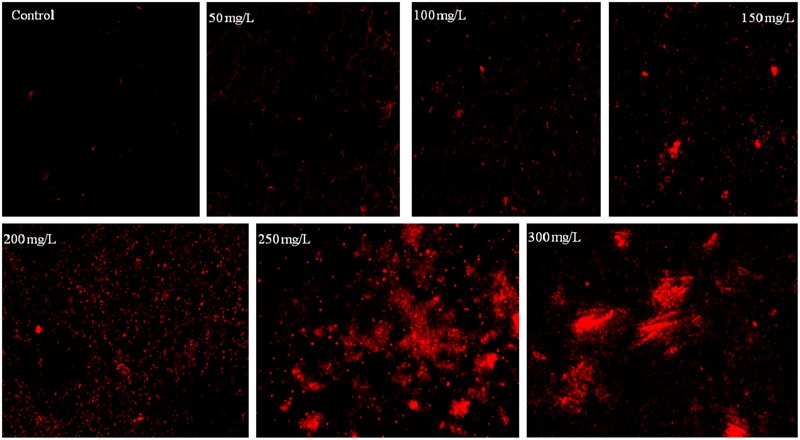
**Fluorescence microscopy images of *B. subtilis* stained with PI incubated at different concentration of chromium**.

**FIGURE 4:**
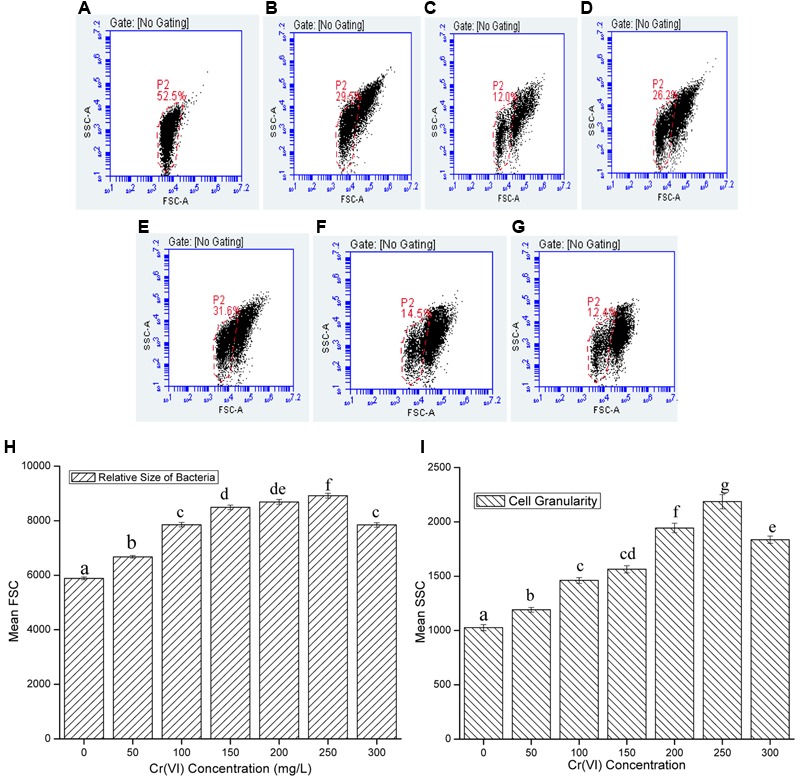
**Representative dot-plots of cell size (FSC) and cell complexity (SSC) at various concentration of chromium (A)** 0 mg/L; **(B)** 50 mg/L; **(C)** 100 mg/L; **(D)** 150 mg/L; **(E)** 200 mg/L; **(F)** 250 mg/L; **(G)** 300 mg/L; and **(H)** relative size and **(I)** granularity of *B. subtilis* MNU16 at different Cr(VI) concentrations. Data are average ± standard deviation of experiments in triplicate. Bars followed by various letter(s) showed significant difference at *P* < 0.05 significance level according to single factor ANOVA.

**FIGURE 5:**
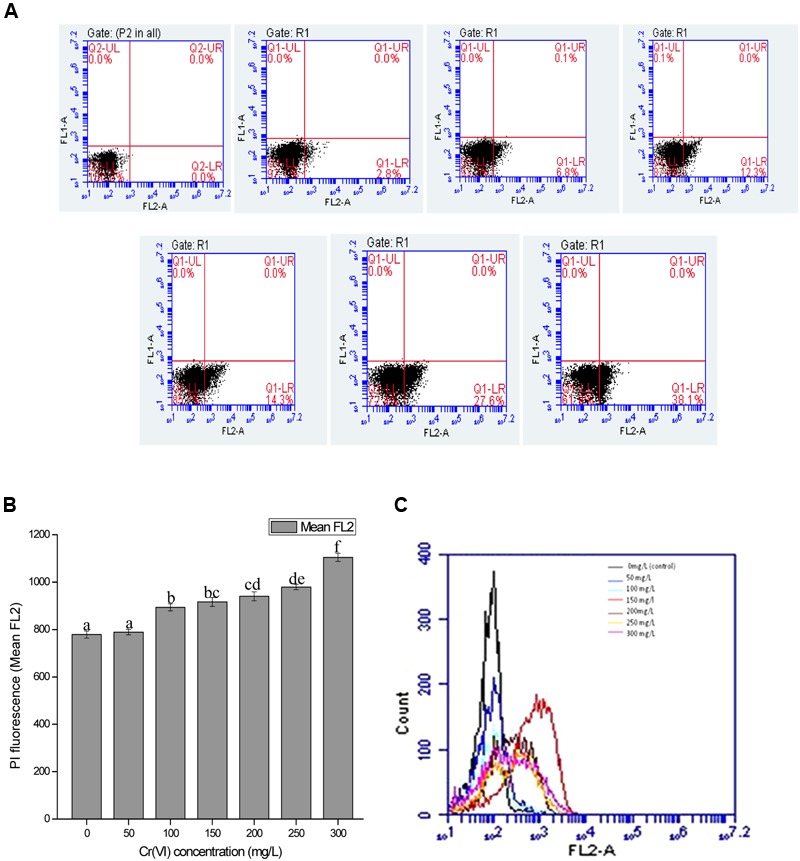
**(A)** Graph represents dot plots of mean FL2-A which shows the PI inclusion (% cell death) at various chromium (VI) concentrations ranged from 0 mg/L (control), 50, 100, 150, 200, 250, and 300 mg/L. **(B)** Overview of changes in cell membrane permeability (PI fluorescence) with increasing Cr(VI) concentration **(C)** Overlay graph of cell count versus PI fluorescence with increasing Cr(VI) concentration. Data are average ± standard deviation of experiments in triplicate. Bars followed by various letter(s) showed significant difference at *P* < 0.05 significance level according to single factor ANOVA.

Bacterial growth was observed in the presence of Cr stress by studying the plate count and growth profile of isolate (**Figure 1E**). The overnight grown bacterial culture was inoculated into the medium having 0 mg/L (control), 50, 100, 150, 200, 250, and 300 mg/L Cr(VI) concentrations, respectively, and was plated onto the nutrient agar medium after 12 h of incubation. The plate count assessment is considered as an initial screening of bacterial viability and the result showed that some morphological changes in bacterial cells were observed upon spreading the bacterial culture after metal exposure. The CFU count was also recorded highest in medium without metal concentration (control) and found to decrease with increasing metal concentration. The results showed that cells have lost their viability in presence of metal concentration which is in agreement with the results of FCM. The bacterial colony was small, pin point, circular at 0, 50, and 100 mg/L chromium concentration, whereas at 150, 200, 250, and 300 mg/L chromium concentration, the colonies were observed to be little elevated and whitish-yellow. It was also concluded from the study that less toxic effect of metal was observed at 50 and 100 mg/L resulting in alteration in colony morphology of bacteria at higher metal concentration. In addition, the physiological changes in bacteria were also studied by TEM (**Figure 2**), FM (**Figure 3**), and FCM in the presence of different chromium treatments (**Figures 4, 5**).

### Transmission Microscopy of *Bacillus subtilis* MNU16

To identify the effect of hexavalent chromium inside cells of *B. subtilis* MNU16, TEM analysis was performed. The modifications due to Cr treatments (0, 100, 200, and 300 mg/L) in bacterial cells have been indicated by pointing out the arrow in the **Figures 2A–D**. The results signified that in control where no Cr treatment was provided, the bacterial cells have a smooth surface (**Figure 2A**). Modification in cell surface were recorded with increasing concentration of Cr(VI) (**Figures 2B–D**). Results further suggested that, once the bacterial cells get exposed in the presence of Cr(VI), the size of bacterium increased and an irregular shape and rough surface were observed as compared to control cells. The effect of Cr(VI) at high concentration (300 mg/L) initiates cell lysis which might be due to the deposition of chromium in the cytoplasm and on the surface of the cells (**Figures 2C,D**).

### Cell Viability Assessment by Fluorescence Microscopy

The bacterium *B. subtilis* MNU16 was subjected to different chromium treatments (Supplementary Figure S2). The bacterial viability was assessed qualitatively using FM by staining bacterial cells with PI. PI is most often used as cell death marker due to its exclusion by the cell membrane of live cells; therefore, the fluorescence bestowed by the dye is usually related to cells whose membrane integrity was damaged. The PI inclusion by bacterial cells shows the percentage of bacterial death at different stress conditions. When the cells were stained with PI (red fluorescence), dead cells acquired the stain and appeared as red color spots when observed by FM (**Figure 3**). Results shows the images of bacterial isolates exposed to various concentrations of chromium ranged from 50 to 300 mg/L, as observed by FM. It is clearly observed from the result that very few populations of bacterial cells were stained in control; however, the population of stained cells increased with increasing concentrations of chromium (**Figure 3**).

### Effect of Cr Treatments on Cell Size and Granularity of *B. subtilis*

Chromium is used to generate stress in bacterial cells and the effect of chromium at various concentrations on cell size and granularity is shown in **Figure 4**. It was observed that by increasing concentration of metal, cell size and granularity of bacteria increases as shown in the figure below (**Figure 4**). The dot plot of *B. subtilis* MNU16 in the presence of chromium(VI) treatment at various concentrations shows that with increasing the concentration of metal, a shift in population was observed and clearly visible at concentration 100, 150, 200, 250, and 300 mg/L defining the changes in FSC and SSC; therefore, it is concluded that the relative size and granularity of the bacterial cells was affected due to the chromium treatment (**Figures 4A–G**).

### Effect of Cr Treatments on Membrane Integrity and Cell Viability of *B. subtilis*

The cell membrane of all the microorganisms allows the cells to interact with the surrounding environment. The cell membrane is selectively permeable and controls the transport of molecules within the cell. To quantify or visualize dead cells by studying the membrane integrity of cells, nucleic acid stains are most commonly used because cell contains relatively high percentage of nucleic acids. Therefore, dead cells acquires the stain by binding with nucleic acids and shows enhancement in fluorescence which aids in quantifying the cell viability during stressed environment. The membrane integrity of *B. subtilis* MNU16 was checked by capturing fluorescently stained cells with PI. In current study, PI was used to discriminate between healthy stable cells and cells with ruptured membrane. PI, a small hydrophilic molecule, is impermeable to the membrane of viable cells, therefore excluded by viable cells and penetrates the membrane of dead cells having damaged membranes and intercalates double stranded DNA and emits fluorescence. It was observed that the mean fluorescence intensity is higher for the cells having ruptured membrane which is proved to be beneficial for a proper discrimination of dead and viable cells in FCM.

In the present study, cells when treated with chromium give high PI fluorescence intensities indicating the percentage cell death in the presence of chromium. Respective dot plots showing the percentage cell death in bacterial cells after Cr(VI) treatment are presented in **Figure 5A**. When bacterial cells are treated with chromium salt, the cytoplasmic membrane potential (represents the difference of electric potential between inside and outside of cell) may get affected and the cytoplasmic membrane permeability gets destroyed. Passive and active transport systems across the cytoplasmic membrane of healthy bacterial cells generate an electrochemical gradient, giving a measure of cellular metabolic activity. Very less intensity was observed at 50 mg/L concentration, whereas a significant increase was recorded with increasing metal concentration. However, at concentration 300 mg/L, the intensity was reduced a bit as compared to low metal concentration. The percentage of cell death (**Figure 5A**), the relative PI fluorescence (**Figure 5B**) and cell count versus relative PI fluorescence graph (**Figure 5C**) recorded in the cells in control and treatments are shown and it is concluded that resistance pattern of bacteria increases with increasing chromium concentration. In control, gaiting was done in a way so that no cell death is recorded and in comparison with the control, least cell death (2.8%) (**Figure 5A**) was recorded in 50 mg/L Cr(VI) concentration and cells that were exposed to highest concentration of chromium gave maximum fluorescence with highest percentage of cell death (38.1%) (**Figure 5A**) indicating the maximum toxic effect of chromium at 300 mg/L; however, percentage cell death was approximately low in the concentration 50, 100, and 200 mg/L (**Figure 5A**) and increases above 200 mg/L Cr(VI) concentration.

## Discussion

Contamination of soil with chromium can modulate the soil microbial diversity and metabolic activity. In addition, nutrient deficiency was also reported in metal contaminated soils which often causes stress for plants (Qishlaqi and Moore, 2007; Ladwani et al., 2012). Till now, several PGP rhizobacteria have been identified such as *Azotobacter, Bacillus, Pseudomonas, Azospirillum*, and *Serratia* for their potential to enhance plant growth by solubilizing minerals, producing iron chelating compounds (siderophores), phytohormones and biocontrol compounds and enhancing phytoremediation of metal contaminants (Hansda et al., 2014; Ndeddy Aka and Babalola, 2016; Sarathambal et al., 2017). Chromium [Cr(VI)] exerts toxic effect on microorganisms by different mechanisms. Chromium resistant bacteria can overcome toxic effects of Cr(VI) and have been utilized for bioremediation of contaminated sites (Samuel et al., 2013; Sobariu et al., 2016). It has been reported that several metal resistant PGP rhizobacteria enhance metal translocation and improve metal uptake efficiency (Hansda et al., 2014).

Isolation and utilization of PGP rhizobacteria for rhizoremediation of heavy metal contaminated soils have gained worldwide attention because it is an environmental friendly and cost-effective approach. Therefore, the current study deals with the isolation and characterization of chromium-resistant-PGP bacteria capable of reducing Cr(VI) to Cr(III). The PGP characteristics were screened and the potential PGP isolate was further screened for chromium resistance and tolerance potential. The reduction of Cr(VI) to Cr(III) was also studied and subsequently the morphological and physiological responses were checked to study the resistant pattern of bacterial strain in the presence of various chromium concentrations to evaluate its impact on bioremediation potential.

The results of the present study confirm those from previously reported literature on *Bacillus* sp. regarding its ability to produce PGP substances and promote plant growth (Shukla et al., 2012; Cherif-Silini et al., 2013; Ndeddy Aka and Babalola, 2016). Production of a plant growth regulator, such as IAA, is an important characteristic of PGP rhizobacteria (Saharan and Nehra, 2011). In the present study, production of IAA was reported by the test isolate and the concentration of IAA (**Table 1**) produced was in agreement with other studies (Karnwal, 2009; Shukla et al., 2012). However, the intrinsic potential of bacteria to produce growth hormone such as IAA in the plant rhizosphere greatly relies on the accessibility of precursors and uptake of microbial IAA by plant (Arshad and Frankenberger, 1991).

The potential of the bacteria to solubilize the insoluble tricalcium phosphate *in vitro* is an additional significant method for accomplishing plant growth promotion (Saharan and Nehra, 2011). The present study shows that the isolate MNU16 solubilizes maximum phosphate (285.13 ± 1.05 μg/ml) after 120 h of incubation, and with increasing incubation time, the pH of the medium declined to 4.1 from an initial pH of 6.4 as shown in **Figures 1A,B**. The result draws support from the previous studies which highlighted the solubilization of phosphate by producing several types of organic acids by different *Bacillus* species and other bacterial species along with the subsequent drop in medium pH (Shukla et al., 2012; Cherif-Silini et al., 2013). Besides, phosphate solubilization and IAA production, the isolate possess another important characteristic of PGP activity, i.e., ACC deaminase activity, in the bacterial isolates which are known to mitigate plant stress by reducing ethylene concentration in plants (Karthikeyan et al., 2012). ACC deaminase activity is an important property that diminishes plant stress caused by adverse environmental stresses such as presence of high metal concentration. ACC deaminase converts ACC, which serves as an ethylene precursor in plants, to ammonia and α- ketobutyrate and thus reduces the plant stress caused by higher ethylene concentration (Karthikeyan et al., 2012). A significant concentration of α- ketobutyrate was produced by the selected isolate in the present study (**Table 1**). It proves that bacteria possess the attribute of phytoremediation by facilitating plants to attain higher and dense roots in chromium contaminated soils.

Siderophore molecules exert both growth promotion and biocontrol mechanisms and have significantly high affinity for iron (Saharan and Nehra, 2011). Consequently, the low availability of iron in the surrounding environment of plant rhizosphere could suppress the growth of pathogenic microorganisms (Patil et al., 2014). In the present study, the bacterium isolated shows a highly orange halo zone in plate assay which confirms siderophore production. Therefore, it was studied quantitatively for estimation of siderophore, which was significantly lower than the values previously reported for PGP strains (Patil et al., 2014; Dutta et al., 2015).

The MIC of the bacteria for chromium was obtained by agar well diffusion method in Muller Hinton agar medium with Cr(VI) concentration ranging from 50 to 1000 mg/L and the bacterial isolate MNU16 was found to be highly resistant for chromium with a concentration of 900 mg/L (Supplementary Figure S1). In addition, the present isolate was also found to be resistant for various other metals (Supplementary Figure S1). The previous studies reported the tolerance range of chromium resistant microorganism from 100 to 4000 mg/L Cr(VI) (Thacker et al., 2007; Zhu et al., 2008). The isolated *Bacillus* sp. MNU16 falls within the above reported range of tolerance. From the molecular characterization and phylogenetic analysis of the test isolate, it was found that the test isolate MNU16 showed maximum similarity with the *B. subtilis* species (**Figure 1C**). Not many isolates from the genus *Bacillus* have been known to tolerate and reduce Cr (VI) (Ibrahim et al., 2011; Mangaiyarkarasi et al., 2011). This difference in resistance profile may be because of the variation in medium composition used for studying the chromium toxicity which influences the toxicity via ‘masking’ effect (Caravelli et al., 2008).

In the present study, *B. subtilis* MNU16 shows chromium reduction to a concentration of 13.23 mg/L (**Figure 1D**) from an initial concentration of 50 mg/L within 72 h of incubation. The reduction efficiency of Cr(VI) for *Bacillus* sp. MNU16 was evaluated and shown in **Figure 1D** and the percentage (%) reduction of Cr(VI) was recorded upto 75% by the bacterium *Bacillus* sp. MNU16 (**Table 1**). The results are in accordance with the previous study which also showed reduction of chromium by *Bacillus* sp. (Samuel et al., 2013) and the Cr(VI) reduction percentages lies within the previously reported studies (Maqbool et al., 2015). This inherent ability of many bacterial species to resist and reduce chromate both under field and laboratory conditions have been previously studied for remediation of chromium contaminated environments (Ibrahim et al., 2011; Maqbool et al., 2015). However, the exact mechanism that is responsible for the reduction of Cr(VI) to Cr(III) is not completely known, and various explanations have been given previously (Ahemad, 2015). The possible mechanism behind the conversion of Cr(VI) to Cr(III) can be either when chromate serve as a final electron acceptor to acquire energy, or the bacteria may secrete some waste product which can possibly reduce Cr(VI) to Cr(III) or by the secretion of several enzymes that might act on Cr(VI) and reduce its toxicity by converting it into Cr(III) (Ahemad, 2015).

Microorganisms when subjected to heavy metals become susceptible to the toxicity caused by reactive oxygen species. Due to the PGP and metal resistance properties, the isolates can be used to remediate metal contaminated soil. The PGP activities of microorganisms are one of the reasons behind the survival of microorganism in metal contaminated soils. The siderophore producing microorganisms have the potential to bind with metals present in surrounding and therefore enhance the bioavailability of metals in plant rhizosphere (Sarathambal et al., 2017). In general, the phytoremediation process of contaminated soils will enhance with the increase in plant growth and metal bioavailability (Ahemad, 2015).

Before studying the physiological response of *Bacillus* sp. MNU16 by FCM, the bacterial response in the presence of Cr(VI) was also studied in the liquid medium by studying the growth pattern of isolate under various chromium concentrations. The growth profile of *Bacillus* sp. MNU16 is shown in **Figure 1E**. It was clearly observed from growth profile (**Figure 1E**) that the absorbance at 300 mg/L chromium concentration was less which indicated that chromium showed highest toxicity at concentration 300 mg/L as compared to other concentrations. The toxic effect of metal was effective in the liquid medium (Shakoori et al., 2000) as the mobility of metals is comparatively more in liquid medium as compared to solid medium. The reduced growth observed in the presence of higher concentrations of Cr(VI) was attributed to the reduction of metabolic rate of bacterium by Cr(VI) (Pal and Paul, 2004).

The application of electron microscopy to examine the morphology of cells and modification in cell structure due to exposure to various contaminants can provide novel dimensions. The results obtained from TEM analysis of *B. subtilis* MNU16 demonstrated the allocation of FDP inside and outside cells which might be due to the exposure of hexavalent chromium treatments. Similarly, study conducted by Batool et al. (2014) reported similar pattern of FDP and cell disruption in cells of *P. aeruginosa* and *Ochrobactrum intermedium* treated with different concentrations of Cr(VI). Further, in the present study, the deposition of EDP inside the *B. subtilis* MNU16 might be due to the accumulation and intracellular reduction of Cr(VI) as shown in **Figures 2C,D**. The study of TEM also showed that the toxicity of Cr(VI) is maximum at 300 mg/L Cr(VI) concentration as shown in **Figure 2D**. The intracellular reduction mechanism of Cr(VI) was reported previously on the strains of *Acinetobacter* sp. and *Shewanella oneidensis* which explains the deposition of EDP inside the cells (Daulton et al., 2007; Srivastava and Thakur, 2007). Thus the presence of these EDP inside cells clearly revealed the modification mechanisms of Cr(VI) into Cr(III) in the form of hydroxyl and carboxyl groups (Bruins et al., 2000; Bencheikh et al., 2007).

The resistance pattern of the test isolate *Bacillus* sp. MNU16 was further studied by the flow cytometric technique in the presence of different chromium concentrations. FCM method has been previously studied for analyzing bacterial physiology when exposed to various stresses (Ionescu et al., 2015; Olszewska et al., 2016) as well as for screening antibiotic susceptibility in bacteria and fungi (Wright et al., 2006). This technique is more suitable for studying physical and chemical characteristics of bacteria due to its ability to rapidly and efficiently analyze large number of microorganisms with better accuracy. The current study reports the use of PI, an exclusion dye used for the analysis of cell viability and complexity by FCM analysis of *B. subtilis* MNU16, during chromium treatments to evaluate its effect on the membrane permeability and eventually on cell viability. The relative sizes and internal complexity of bacterial cells were estimated by flow cytometric analysis by estimating the forward scatter (FSC) and side scatter (SSC) of light, respectively, and shown in **Figures 4A–I**. The results showed that with increasing chromium concentration, a shift in FSC and SSC of bacteria was observed (**Figure 4**). The forward scatter (FSC) is affected by the size of bacteria and the intensity of scatter depends on the refractive index of the particle and flow medium; although, for particles having size in the range of bacteria were found to be correlated with the total volume of bacterial cell (Baatout et al., 2007). The side scatter of light (SSC) is proportional to cell granularity or internal complexity. The reflected and refracted light was measured at several interfaces of bacterial cell where alteration in refractive index was observed and also aids in detecting the presence of inclusion bodies generated due to the environmental stresses (Baatout et al., 2007).

The mean FL2 graph showing the relative fluorescence due to PI staining of *B. subtilis* MNU16 grown in the presence of various chromium concentration given in **Figure 5B**, describing the comparison of cell count and fluorescence intensity at various Cr(VI) concentrations. It is clear from the **Figure 5C** that high cell count and less fluorescence intensity was observed in the control sample where no stress was provided and eventually a decrease in cell count was recorded when growing the cells in the presence of different Cr(VI) concentrations.

The study shows that a significant population of bacterial species is present in the medium at high concentration of chromium which shows *B. subtilis* MNU16 has chromium resistance potential. The noticeable increase in PI fluorescence intensity with increasing Cr(VI) concentration (**Figure 5**) shows the enhancement in loss of membrane integrity with the increasing concentration of chromium. Furthermore, in the presence of chromium treatments, variation in cell death was observed by varying the concentration of chromium with the help of FM. The presence of maximum number of cells stained with PI showed the highest cell death in the sample which is correlated with the data obtained from the PI florescence of FCM as shown in **Figure 5**. These results are confirmed by the data obtained by florescence imaging of bacteria that gives fluorescence when PI intercalates with bacterial DNA which shows the increase in membrane permeability due to membrane damage.

Furthermore, the results obtained from the study confirm the fact that the most crucial target for any environment stress is cell membrane whether it is pH, heavy metal or temperature (Mangaiyarkarasi et al., 2011; Olszewska et al., 2016). This suggests that differences in properties of cell membrane can possibly be an essential factor in establishing the metal tolerance potential between various bacterial strains. On the other hand, the nature of Cr(VI) toxicity as a function of membrane composition and structure in determining tolerance of metal and other environmental stresses still needs to be clarified.

## Conclusion

The main focus of the study was to isolate an efficient chromium resistant PGP rhizobacterium having the potential to tolerate chromium stress which can be utilized for bioremediation purpose. The selected *B. subtilis* MNU16 strain shows significant PGP characteristics highlighting resistance to high concentration of chromium and able to decrease the toxicity of Cr(VI) by reducing it to Cr(III), a less toxic form. The resistance pattern against chromium was studied by analyzing the cell viability percentage by FCM and FM. FCM presents the probability of performing both qualitative and quantitative evaluation established on the simultaneous measurements of structural and practical parameters of single cell. The study concluded that the isolated PGP rhizobacterial strain with significant Cr(VI) reduction potential provides new possibilities to design strategies for improving the efficacy of rhizoremediation (microbial assisted phytoremediation) of contaminated soils.

## Author Contributions

NU, KV, JS, RM, SS, MM, and DT designed experiments. NU, KV, RM, and JS performed experiments. NU, KV, VK, RR, DC, RM, and DT, analyzed data and wrote the manuscript. DT, DC, and SS critically evaluated the manuscript.

## Conflict of Interest Statement

The authors declare that the research was conducted in the absence of any commercial or financial relationships that could be construed as a potential conflict of interest.

## Abbreviations

CFU: colony forming unit
EDP: electron dense precipitates
FCM: flow cytometry
FM: fluorescence microscopy
MHA: Mueller Hinton Agar
MIC: minimal inhibitory concentration
PI: propidium Iodide
TEM: transmission electron microscopy
