# Supplementary material for: Tolerance and Reduction of Chromium(VI) by Bacillus sp. MNU16 Isolated from Contaminated Coal Mining Soil
Source: Front Plant Sci. 2017 May 22;8:778. doi: 10.3389/fpls.2017.00778 (PMC5438964; doi:10.3389/fpls.2017.00778)
Supplement: Supplementary file 1 [file Image_1.PDF]

Figure S1: Minimal inhibitory concentration (MIC) of *Bacillus* sp. MNU16 against heavy metals (a) Chromium (b) Mercury (c) Iron (d) Cadmium (e) Arsenic and (f) Nickel

Figure S2: Growth of *Bacillus* sp. MNU16 in nutrient broth medium amended with different chromium concentration ranged from 0 mg/L to 300 mg/L

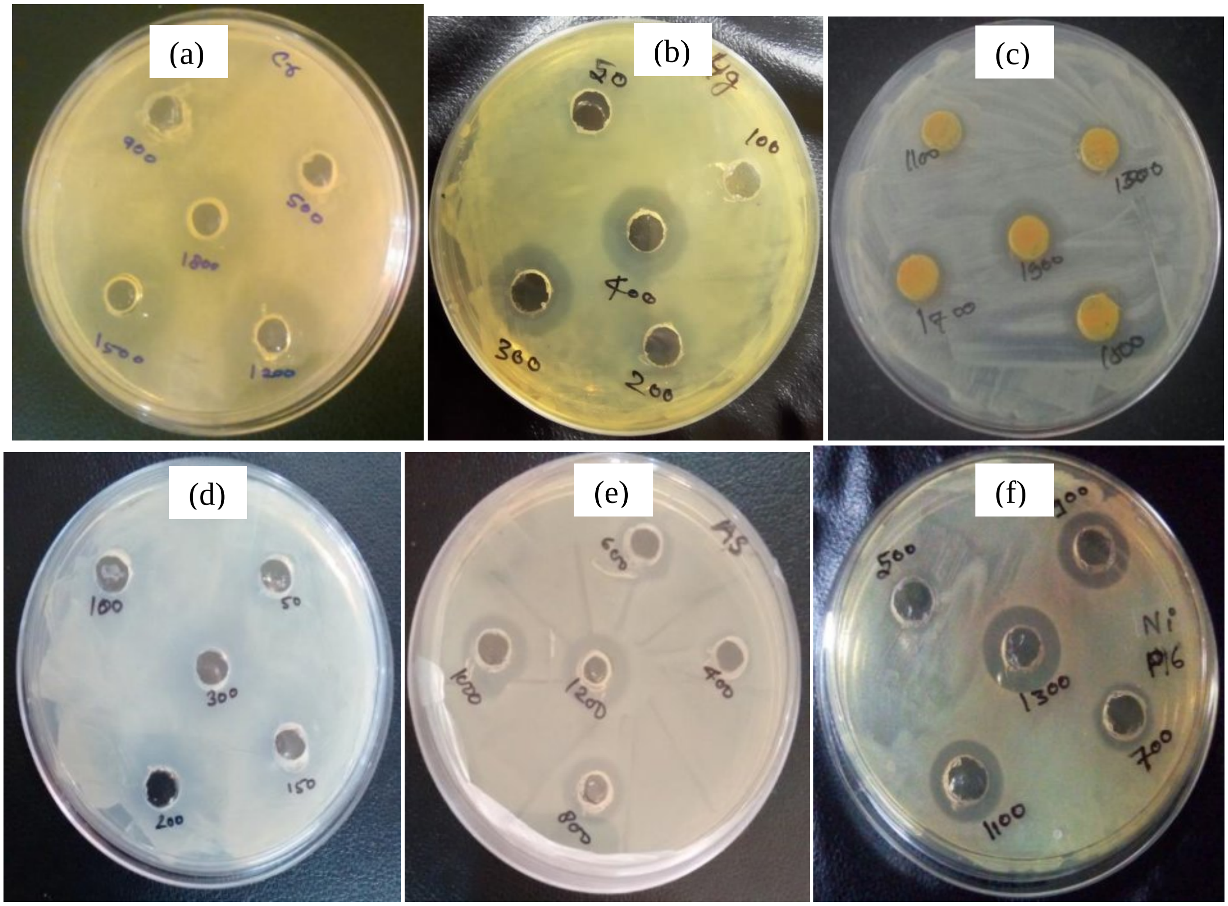
(a)
(b)
(c)
(d)
(e)
(f)

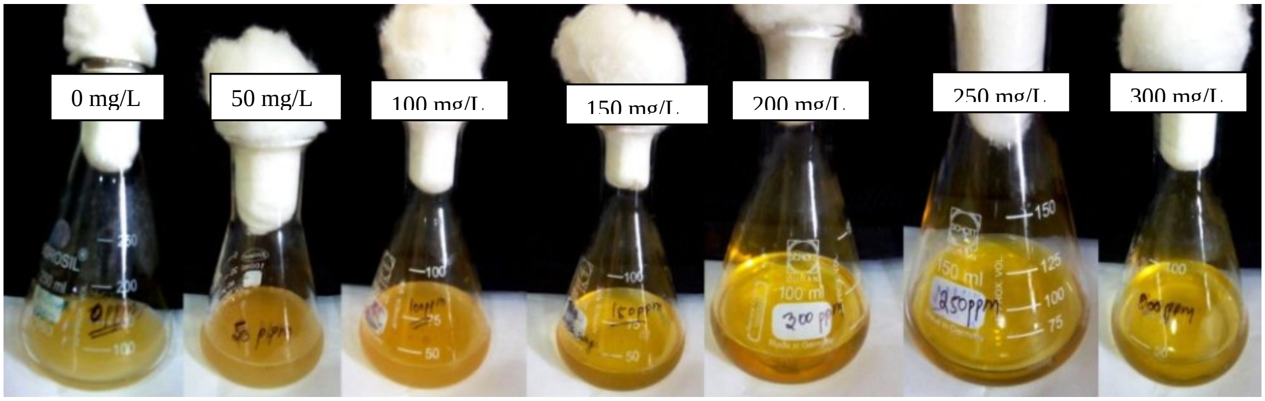
0 mg/L
50 mg/L
100 mg/L
150 mg/L
200 mg/L
250 mg/L
300 mg/L
